# Knowledge and Attitude Towards Cutaneous Complications of Tattoos and Piercings Among the General Population in Saudi Arabia

**DOI:** 10.7759/cureus.49912

**Published:** 2023-12-04

**Authors:** Lina I Kinkar, Maan A Magboul, Ghadeer E Alamri, Esraa A Shaheen, Elaf R Altalhi, Abdullah M Alharthi, Ahmed M Baabdullah

**Affiliations:** 1 Department of Medicine, College of Medicine, Umm Al-Qura University, Makkah, SAU; 2 Department of Medicine, King Abdulaziz University Faculty of Medicine, Jeddah, SAU; 3 Department of Medicine, King Abdullah International Medical Research Center, Jeddah, SAU; 4 Department of Medicine, King Saud Bin Abdulaziz University for Health Sciences College of Medicine, Jeddah, SAU; 5 Department of Dermatology, King Abdulaziz Hospital, Makkah, SAU; 6 Department of Dermatology, King Abdulaziz University Hospital, Jeddah, SAU; 7 Department of Dermatology, King Abdulaziz University Faculty of Medicine, Jeddah, SAU

**Keywords:** health risks, practice, koweldge, body arts, body piercing, tattoo

## Abstract

Introduction

Tattooing and piercing, which were uncommon in affluent countries, have recently spread throughout societies. Over the past two decades, young people have shown considerable excitement for the practice. This reported growth creates more difficulties because of the numerous operations performed without awareness of health and hygiene requirements. This study aims to assess the knowledge and attitudes of the general population toward the cutaneous complications of tattoos and piercings in Saudi Arabia.

Methods

This was a web-based descriptive cross-sectional study. An online questionnaire was developed by the study researchers, which included participants’ demographic data, their knowledge of tattoos and piercings with associated health effects and possible infections, their attitude towards tattoos and piercings, and their practice and experienced complications regarding tattooing and body piercing. The final validated questionnaire was made publicly available via social media until no more new responses were obtained.

Results

Eight hundred and forty-eight individuals completed the study questionnaire. The ages of the participants varied from 18 to more than 55 years old, with a mean age of 25.2 ± 13.9 years, and (589 [69.5%]) were female. The most reported health effects and infections were bacterial infections in place of body modification (479 [56.5%]), purpura (380 [44.9%]), cutaneous abscesses (380 [44%]), melanoma (338 [39.9%]), hepatitis B virus (321 [37.9%]), HIV (311 [36.7%]), sepsis (306 [36.2%]), allergic contact dermatitis (296 [35%]), and hepatitis C virus (279 [33.1%]). In total, 336 (39.6%) had an overall good knowledge of tattooing and body piercing hazards, with higher knowledge among females with high education (p< 0.05).

Conclusion

This study revealed that one in three individuals knew about the health risks and infections associated with tattooing and body piercing. Higher awareness was found among females with high education levels. Of all the associated complications, procedure-related infections were the most common among participants.

## Introduction

Body modification, also known as tattooing or skin piercing, is now a dominant practice, especially in modern cultures. The growing incidence of people opting for ink body painting has attracted the attention of academics worldwide [1,2]. However, this growing demand has increased the number of unprofessional body art practitioners performing procedures that require more adherence to safe and healthy hygienic standards [3]. Consequently, the frequency of recorded adverse health hazards after tattooing has increased [4]. The prevalence of cutaneous complications of tattoos ranges from 2%-43% [5,1,6], even though the cutaneous complications are higher than the systemic complications. A survey of 3411 participants showed that 2302 (67.5%) developed skin problems, while only 225 (6.6%) reported extracutaneous problems [1].

Tattooing may cause substantial dermatological complications, including rash due to hypersensitivity reactions, allergic contact dermatitis, lichenoid, granulomatous cutaneous disease, and the formation of hypertrophic scars and keloid [7]. Furthermore, cutaneous infectious complications may progress rapidly after the tattooing procedure, such as viral infections (verruca vulgaris, molluscumcontagiosum, hepatitis B virus [HBV] and C virus [HCV], herpes simplex, or infection with human immunodeficiency virus [HIV]), bacterial infections (*Streptococcus*, *Staphylococcus*, *Pseudomonas aeruginosa*, cutaneous tuberculosis, syphilis, pyoderma, and dysbacteriosis), and fungal infections (dermatophytosis and sporotrichosis) [7-11]. However, allergic and photoallergic reactions, infections, and the Koebner phenomenon are the most commonly reported consequences [12].

Despite the vast research assessing the knowledge of the global complications of tattooing and piercing, little is known about the topic in Saudi Arabia. Therefore, this study evaluated the knowledge and attitudes about the cutaneous complications of tattooing and piercing among Saudi people.

## Materials and methods

A descriptive cross-sectional web-based study was conducted in Saudi Arabia to assess public knowledge, behavior, and practice toward tattoos and piercings. All individuals over the age of 18 who consented to participate in the study were included in the final analysis. The study excluded individuals who refused to participate, were younger than 18 years old, or spoke a language other than Arabic. The study group was determined by a simple random method.

All participants consented before filling out the survey, and all information was kept completely confidential in accordance with the ethical approval received from the institutional review boards (IRB) for our study. The study investigators created an online questionnaire based on a review of the relevant literature and consultations with two dermatology consultants [6-13]. The validity, applicability, and clarity of the questionnaire were evaluated independently by three experts, and all modifications were made to obtain the final version. The survey was prepared in English and translated into Arabic. An Arabic copy was distributed to the participants. Then, the Arabic version was translated back to English by bilingual experts to ensure consistency.

The anonymous questionnaire was published on social media platforms from March 27 to April 10, 2023. Clarifications regarding the extent of confidentiality and the significance of this research to society’s health were used to encourage respondents to participate. This study questionnaire included participants’ demographic information (age, gender, geographic location, education, occupation, and marital status); knowledge of tattoos and piercings, their associated health effects, and possible infections; attitudes towards tattoos and piercings, assessed using a 5-point Likert scale; and evaluated participants’ practice and whether they experienced complications regarding tattooing and body piercing. The final, approved survey was distributed across social media platforms until no more replies were received.

Data were collected and analyzed using the IBM Corp. Released 2012. IBM SPSS Statistics for Windows, Version 21.0. Armonk, NY: IBM Corp. All the statistical methods used were two-tailed with an alpha level of 0.05, and a p-value < 0.05 was considered significant. The overall level of knowledge about tattoos and piercings and the health risks was measured by adding the scores for the different awareness items. If a participant’s overall knowledge scores < 60% of the total score were considered a bad level of knowledge, scores ≥ 60% of the total score were considered a good level of knowledge. Frequencies and percentages were used to describe study variables, such as personal information, residence area, and occupation of participants. Participants’ knowledge, attitudes, and behavior concerning tattoos and piercings with associated health hazards were tabulated, while overall knowledge and reported adverse health effects and possible infections were presented using plots. Using a Pearson chi-square test for independence and an exact probability measure, slight variations in frequency cross-tabulation were performed to identify factors associated with study participants’ awareness of the health hazards associated with tattoos and piercings.

## Results

In total, 848 participants completed the survey with a response rate of 98%. Twelve participants refused to complete the survey: 257 (30.3%) were from the western area, 201 (23.7%) were from the central area, 175 (20.9%) were from the eastern area, 111 (13.1%) were from the southern area, and 104 (12.0%) were from the northern area, respectively. The ages of the participants ranged from 18 to over 55 years old, with an average age of 25.2 ± 13.9 years. Regarding the level of education, 570 (67.2%) had a bachelor's degree, 149 (17.6%) had secondary education or less, and 60 (7.1%) had a postgraduate degree. A total of 441 (52%) were students, and 253 (29.8%) were employed (Table 1).

**Table 1:** Socio-demographic data of the study participants

Socio-demographic data	No	%
Region		
Central	201	23.7%
Northern	104	12.3%
Eastern	175	20.6%
Western	257	30.3%
Southern	111	13.1%
Age in years		
18–25	505	59.6%
26–35	195	23.0%
36–45	68	8.0%
46–55	55	6.5%
> 55	25	2.9%
Gender		
Male	259	30.5%
Female	589	69.5%
Nationality		
Saudi	793	93.5%
Non-Saudi	55	6.5%
Marital status		
Single	564	66.5%
Married	255	30.1%
Divorced/Widow	29	3.4%
Educational level		
Secondary/Below	149	17.6%
Diploma	69	8.1%
Bachelor	570	67.2%
Postgraduate	60	7.1%
Occupation		
Not working	134	15.8%
Student	441	52.0%
Employee	253	29.8%
Retired	20	2.4%

The knowledge of tattoos and piercings among the general population in Saudi Arabia as demonstrated in Table 2.

**Table 2:** Knowledge of tattoos and piercings among the general population in Saudi Arabia

Knowledge items	No	%
Negative health effects or possible infections during body modifications?		
Yes	593	69.9%
No	93	11.0%
I do not know	162	19.1%
Is it risky undergoing piercing/tattooing?		
Yes	702	82.8%
No	39	4.6%
I do not know	107	12.6%
Can tattoos and piercings transmit infectious disease?		
Yes	581	68.5%
No	51	6.0%
I do not know	216	25.5%
Can tattoos and piercings transmit non-infectious disease?		
Yes	244	28.8%
No	203	23.9%
I do not know	401	47.3%
Are the places and instruments used for body art always safe in terms of health and hygiene?		
Yes	76	9.0%
No	551	65.0%
I do not know	221	26.1%
Is it possible to remove the tattoo?		
Yes	522	61.6%
No	158	18.6%
I do not know	168	19.8%
Is piercing a permanent practice?		
Yes	178	21.0%
No	257	30.3%
I do not know	413	48.7%

Five hundred and ninety-three (69.9%) of the participants knew about negative health effects or possible infections during body modifications; 702 (82.8%) knew that it is risky to undergo piercing or tattooing; 581 (68.5%) knew that tattoos and piercings transmit infectious disease; 244 (28.8%) knew about the possibility of having non-infectious disease due to tattooing; and 79 (9%) were aware that the locations and tools used for body art are always hygienic and safe for health. About 522 (61.6%) knew it was possible to remove the tattoo, while 178 (21%) reported that piercing is a permanent practice.

**Figure 1:**
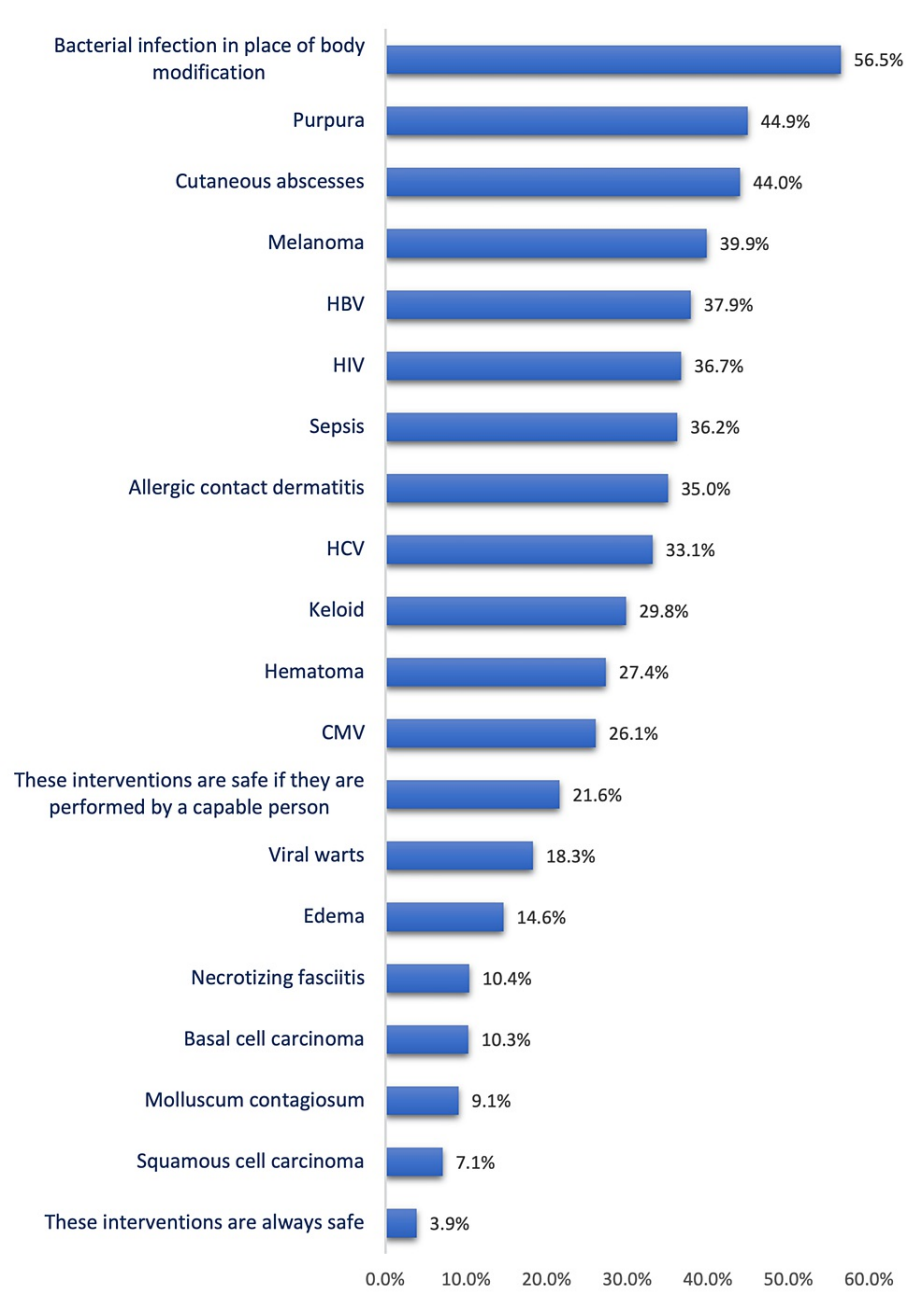
Negative health effects and possible infections during body modifications were reported by study participants. HBV: Hepatitis B virus, HIV: Human immunodeficiency virus, HCV: Hepatitis C virus, CMV: Cytomegalovirus.

The negative health effects and possible infections during body modifications reported by the study participants are shown in Figure 1. The most reported health effects and infections were bacterial infections at the site of body modification (479 [56.5%]), purpura (380 [44.9%]), cutaneous abscesses (380 [44%]), melanoma (338 [39.9%]), HBV (321 [37.9%]), HIV (311 [36.7%]), sepsis (306 [36.2%]), allergic contact dermatitis (296 [35%]), and HCV (280 [33.1%]).

**Figure 2:**
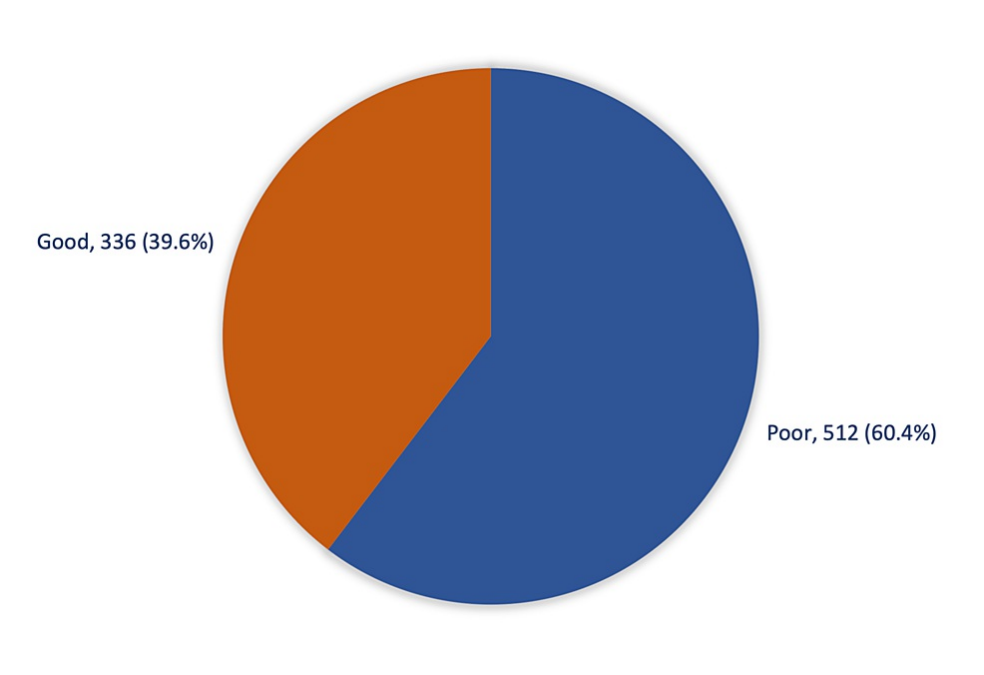
Overall knowledge of tattoos and piercings among the general population in Saudi Arabia

Regarding knowledge of tattoos and piercings among the general population in Saudi Arabia, 336 (39.6%) had an overall good knowledge of tattooing and body piercing hazards (Figure 2).

Regarding participants’ attitudes towards tattoos and piercings, 779 (91.9%) disliked other body modifications, such as subcutaneous implants and a split tongue; 700 (82.5%) disliked tattoos in general; 683 (80.5%) disliked piercings in places other than the ears; and only 279 (32.9%) disliked ear piercings (Table 3).

**Table 3:** Attitude towards tattoos and piercings among the general population in Saudi Arabia

Attitude items	I definitely do not like it		I do not like it		I do not know		I like it		I definitely like it	
	No	%	No	%	No	%	No	%	No	%
What is your opinion about tattoos?	549	64.7%	151	17.8%	52	6.1%	67	7.9%	29	3.4%
What is your opinion about ear piercing?	175	20.6%	104	12.3%	74	8.7%	304	35.8%	191	22.5%
What is your opinion about body piercing in places different other than ears?	476	56.1%	207	24.4%	70	8.3%	68	8.0%	27	3.2%
What is your opinion about other body modifications, such as subcutaneous implants and split tongue?	635	74.9%	144	17.0%	40	4.7%	22	2.6%	7	.8%

Regarding tattoos and piercing practices, only 69 (8.1%) of the study participants reported being pierced anywhere except the ear, and 132 (15.6%) reported considering it in the future. Also, 25 (2.9%) were tattooed, and 59 (7%) considered it in the future. About 143 (16.9%) already possessed prior tattoos or piercings, among whom 43 (30.1%) asked someone’s advice, 80 (55.9%) informed their parents, 57 (39.9%) were informed about the risks related to such practices, and 24 (16.8%) signed any informed consent (Table 4). Among those who received tattoos, 84 (58.7%) were attended by operators using sterile or disposable instruments, while 77 (53.8%) had their tattoos done professionally. Only 25 (17.5%) reported complications after the intervention.

**Table 4:** Practices of tattoos and piercings among the general population in Saudi Arabia

Practice items		No	%
Have you been pierced anywhere except your ear?	Yes	69	8.1%
	No	453	53.4%
	I have not had any piercings before	326	38.4%
If you do not have a piercing, would you consider it in the future?	Yes	132	15.6%
	No	614	72.4%
	I do not know	102	12.0%
Have you been tattooed?	Yes	25	2.9%
	No	528	62.3%
	I have not had any tattoos before	295	34.8%
If you do not have a tattoo, would you consider it in the future?	Yes	59	7.0%
	No	722	85.1%
	I do not know	67	7.9%
Have any tattoos or piercings been done before?	Yes	143	16.9%
	No	705	83.1%
When you decided to undergo body art, did you ask someone’s advice? (n=143)	Yes	43	30.1%
	No	93	65.0%
	I do not know	7	4.9%
Were your parents informed when you underwent body art? (n=143)	Yes	80	55.9%
	No	55	38.5%
	I do not know	8	5.6%
Did you sign any informed consent? (n=143)	Yes	24	16.8%
	No	101	70.6%
	I do not know	18	12.6%
Were you informed about the risks related to such practices? (n=143)	Yes	57	39.9%
	No	75	52.4%
	I do not know	11	7.7%
Did the operator use sterile/disposable instruments? (n=143)	Yes	84	58.7%
	No	25	17.5%
	I do not know	34	23.8%
Did you report any complications after the intervention? (n=143)	Yes	25	17.5%
	No	101	70.6%
	I do not know	17	11.9%
Was the tattoo done in a professional way? (n=143)	Yes	77	53.8%
	No	66	46.2%

Among the factors associated with participants' knowledge of tattoos and piercings, 57 (51.4%) of participants in the southern region had an overall good knowledge of tattoos and piercings and associated health risks versus 26 (25%) of others in the northern region (p = 0.001). Additionally, a higher level of knowledge was observed in females compared to males (42.8% versus 32.4%, p=0.005). Good knowledge was observed among participants with the highest educational level compared to others with diplomas (58.3% versus 14.5%, p = 0.001) (Table 5).

**Table 5:** Factors associated with participants' knowledge about tattoos and piercings

Factors	Overall knowledge level				p-value
	Poor		Good		
	No	%	No	%	
Region					0.001
Central	133	66.2%	68	33.8%	
Northern	78	75.0%	26	25.0%	
Eastern	104	59.4%	71	40.6%	
Western	143	55.6%	114	44.4%	
Southern	54	48.6%	57	51.4%	
Age in years					0.170
18–25	313	62.0%	192	38.0%	
26–35	106	54.4%	89	45.6%	
36–45	38	55.9%	30	44.1%	
46–55	38	69.1%	17	30.9%	
> 55	17	68.0%	8	32.0%	
Gender					0.005
Male	175	67.6%	84	32.4%	
Female	337	57.2%	252	42.8%	
Nationality					0.280
Saudi	475	59.9%	318	40.1%	
Non-Saudi	37	67.3%	18	32.7%	
Marital status					0.307
Single	331	58.7%	233	41.3%	
Married	164	64.3%	91	35.7%	
Divorced/Widow	17	58.6%	12	41.4%	
Educational level					0.001
Secondary/Below	95	63.8%	54	36.2%	
Diploma	59	85.5%	10	14.5%	
Bachelor	333	58.4%	237	41.6%	
Postgraduate	25	41.7%	35	58.3%	
Occupation					0.100
Not working	88	65.7%	46	34.3%	
Student	254	57.6%	187	42.4%	
Employee	154	60.9%	99	39.1%	
Retired	16	80.0%	4	20.0%	

## Discussion

Recently, there has been an increase in the frequency of body art usage, accompanied by many hazardous consequences associated with body tattooing and piercing. Tattooing involves injecting ink into the skin to create permanent designs. The process involves puncturing the skin with a needle, which can lead to bleeding, pain, and the risk of infection. Additionally, there is a risk of allergic reactions to the ink or ink pigments [14]. Body piercing involves creating a hole in the body to insert jewelry. Piercings can be done on various body parts, including the ears, nose, tongue, nipple, and belly button. The risks associated with body piercing include infection, bleeding, scarring, and allergic reactions to the jewelry material [15]. Generally, tattooing and body piercing can be safe if trained professionals adhere to proper hygiene practices. It is essential to be aware of the potential risks and to take appropriate care of the tattoo or piercing to minimize the risk of complications.

The present study analyzed the public’s awareness, attitude, and practice of tattooing and body piercing and their associated health risks. Regarding public knowledge, the survey found that more than one-third of the participants had a decent understanding of body arts and related infections. More than two-thirds of participants knew about adverse health effects or possible infections during body modifications; the vast majority knew that it is risky to undergo piercing or tattooing, and about two-thirds reported that tattoos and piercings transmit infectious diseases. However, very few people believe that the places and tools used for body painting are always safe in terms of health and hygiene. About two-thirds knew it was possible to remove the tattoo, while one-fifth reported that piercing is a permanent practice. Knowledge was significantly higher among some residents in the region, particularly females with high education levels. Abimbola O et al. [16], in Nigeria, found that 340 (85%) of young adults knew about tattoos and body piercing, and 125 (31.3%) reported fashion as the main reason for body modifications. Bhurtel A and Mudhol [17] reported that 24 (48%) of study adults had high knowledge of tattoos and body piercings, while 17 (34%) had a low knowledge level. A study conducted in Italy among students concluded that males were less knowledgeable about infectious diseases related to body art and that students with piercings were less likely to seek therapy for medical complications [18]. Ehwarieme TA et al. [19] found that all university students knew tattoos and body piercings could constitute a health risk.

Considering the health risks and associated complications, this study showed that the most reported health effects and infections were bacterial infections at the body modification site, purpura, cutaneous abscesses, melanoma, HBV, HIV, sepsis, allergic contact dermatitis, and HCV. Ehwarieme TA et al. [19] reported that the most associated health risks and infections included HIV/AIDS, tetanus, hepatitis, pruritus (itching), endorsed skin injury (wound), abscess or boils, inflammation, endorsed damage to underlying blood vessels and nerves, and skin allergies. More than half of the participants in their study said that ongoing infections were a health risk that could result from body piercing or tattooing. Another study on high school students reported a high percentage of health complications following body art procedures. The most common adverse events in tattooed students were skin irritation (30 [19%]) and uncharacteristic bleeding (15 [10.1%]). The most frequent complication associated with body piercing was infection at the site and skin irritation [20]. Many people who had piercings or tattoos had not considered the potentially serious health risks and did not know how to check if the procedure followed the appropriate safety requirements [21].

As for participants’ attitudes, the current study showed a negative attitude towards tattooing and body piercing, except for ear piercing. The participants disliked other body modifications, such as subcutaneous implants, split tongues, and tattoos, besides their dislike of piercing in locations other than the ears. Miaodong, in his thesis, showed a similar attitude among public participants towards tattooing and body piercing, except for students [22]. Schorzman, Gold, Down, and Murry [23] showed that many young adults accept body piercings. Considering public practice for tattooing and body piercing, the current study revealed a considerably low percentage reported undergoing tattooing and body piercing, with only a small percentage considering doing so in the future. This can be explained by the public’s acceptable knowledge of the health-related complications and negative attitudes towards these interventions. Among those who underwent tattooing or body piercing, more than half reported that tattooing was done professionally using sterile or disposable instruments with fewer complications.

As this is the first study of its kind conducted in Saudi Arabia, multiple limitations could be addressed in future research. First, data collection was based on a standardized self-administered questionnaire, which may include a selection and recall bias that can affect the results. Second, many cultural variations between the different regions of Saudi Arabia may significantly affect how people perceive body modification and their knowledge about its associated complications. Therefore, more extensive research is necessary to accurately assess the population’s overall knowledge regarding these procedures and the health risks they could cause.

## Conclusions

This study showed that almost one-third of the participants knew about the health risks and infections related to tattoos and body piercings. Higher awareness was found among females with a high education level. Procedure-related infections were the most common among participants among all associated complications. Most participants showed a negative attitude towards tattooing and body piercing, with a low percentage among those who did tattooing. Moreover, involving family members in the decision-making process was uncommon among those who did tattooing, and they were also informed about the potential risk. Professionals who interact directly with the population should approve appropriate preventive measures. Primarily, older people should be assisted in making informed decisions. Comprehensive educational programs that include inherent risks should also be developed for body artists and encourage young adults to consider their choices carefully beforehand.
